# Unilateral elongated styloid process: a case report

**DOI:** 10.1186/1757-1626-2-9135

**Published:** 2009-12-03

**Authors:** George K Paraskevas, Athanasios Raikos, Loukas M Lazos, Panagiotis Kitsoulis

**Affiliations:** 1 Department of Anatomy, Medical Faculty of Aristotle University of Thessaloniki, PO Box 300, Thessaloniki, 54124, Greece

## Abstract

An unusual case of a unilaterally elongated styloid process with a length of 5.8 cm was found on a dry skull of a male cadaver. During his life the subject was complaining for reported ipsilateral otalgia presumably due to nerve compression from the elongated styloid process. The symptomatology appeared by such an anatomical variant as well as relative literature is discussed in this paper.

## Introduction

The styloid process is a thin, cylindrical, sharp osseous process, deriving from the posterior lower surface of the petrosal bone (just anterior to stylomastoid foramen). The process is directed downwards, to the front and slightly to the inside. The apex of the styloid process is connected with the ipsilateral lesser cornu of hyoid bone via stylohyoid ligament. The ligament represents from embryological view the continuation of the processes apex. The entire previous mentioned features constitute the stylohyoid chain. The whole chain derives embryologicaly from four cartilages: tympanohyale, stylohyale, ceratohyale, and hypohyale. The styloid process originates from the second branchial arch [1].

The styloid process in some cases could be long enough to cause symptoms due to compression of surrounding anatomical structures. Early in 1949 Eagle described the homonymous syndrome, characterized by elongated styloid process or ossified stylohyoid ligament [2]. This paper reports a case of unilateral elongated styloid process on a macerated skull from a male cadaver with ipsilateral otalgia during his life.

## Case presentation

**Figure 1:**
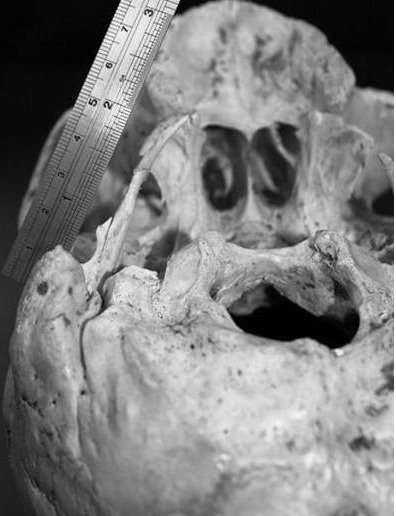
**Posterolateral view of the skull base, in which a right elongated styloid process, is shown**. The flexure observed at the limit of middle and inferior distal 1/3 (arrows) could presumably represent the site of the unification between the apex of the process and the ossified section of the stylohyoid ligament.

**Figure 2:**
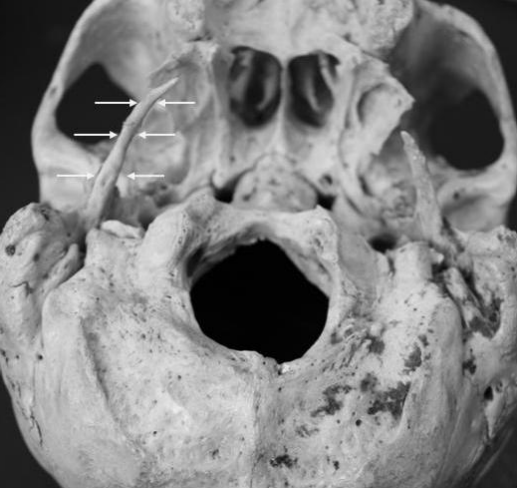
**Posterior-Inferior view of the skull base in which the right styloid process is indicated (arrows)**.

In a dry skull of a male donor cadaver of Caucasian race, aged 72 years old, selected from the osteological collection of our Anatomy Department. From macroscopic analysis an extremely elongated right styloid process was noticed. The measurements were made with the assistance of a digital sliding caliper. The length of the right styloid process was 5.8 cm using as inceptive point the inferior border of the tympanic bone. The left styloid process was 2.3 cm long. The flexure observed at the limit of middle and inferior distal 1/3 could presumably represent the site of the unification between the apex of the process and the ossified section of the stylohyoid ligament (Figure 1 and Figure 2). From the specimens medical history there was no evidence of spondylosis, ankylosing spondylitis or idiopathic skeletal hyperostosis neither any evidence of traumatic lesions at skull base. From case history the specimen was suffering from undiagnosed ipsilateral otalgia. The research done was approved by Ethical Committee of the Aristotle University of Thessaloniki.

## Discussion

Styloid process length ranges from 5 mm to 50 mm [1]. The normal length of the styloid process ranges from 25 mm to 30 mm [2]. Some authors claim that a length between 15.2 mm and 47.7 mm could be considered as normal [3]. Other studies accept that a length of over 30 mm is considered elongated [4,5] and by others 40 mm [4]. Some rare cases exist with length of 73 mm [1]. Incidence seems to range from 1.4 to 84.4% of population, that's due to great variations in radiological methodology and technique, surgical or anatomic (cadaveric or dry) specimens, ethnic variability and predominance of unilateral to bilateral occurrence [6,7]. Sometimes a distinct border between apex and stylohyoid ligament is not clearly identifiable. Styloid process elongation can occur unilaterally or bilaterally. There are investigators claiming that the phenomenon is most common unilaterally [8], but others are contrary to these claims [9].

The first who described a case of stylohyoid ligament ossification seems to be Manchetti of Padua back of 1652 [10]. Diagnosis can be estimated by bimanual palpation of tonsilar fossa (normal styloid process is not normally palpable), panoramic radiography studies and CT. The latter provides additional information to plain radiographs [5].

An elongated styloid process or ossificated stylohyoid ligament is not symptomatic in all cases. Eagle syndrome is always accompanied with symptoms [5]. Those vary from dysphagia, foreign body sensation, throat pain, ipsilateral otalgia, headache, neck pain during rotation, pain during tongue extension, facial and carotid pain [3,11,12].

Moreover, it seems that there is coexistence of ossified stylohyoid ligament with other conditions such as: Cervical osteophytes and cervical spodylosis [11], anomalies in the atlantic section of the vertebral artery [13] and fracture of the ossified ligament [9]. Cervical spondylosis yet very common in elderly population, may have similar clinical signs with Eagles syndrome, but could be differential diagnosed by palpation of tonsilar fossa. Arterial anomalies should be cleared up because it is likely to coexist with stylohyoid ossification. Only 9 cases have been reported in literature with fractures of ossified ligaments. These fractures can be caused spontaneously or traumatic [9,12].

Ossification can take place during childhood and adolescence when the rate of bone growth is increased. After the age of 20 there is a rapid decrease in ossification formation [14]. However, other authors support that an inconsistent trend exists toward greater ossification of the stylohyoid ligament with advanced age [15].

It has been suspected that an elongated styloid process could be caused by: congenital elongation of the styloid process due to persistence of the cartilaginous analog of the Styloid [5], calcification of the stylohyoid ligament by unknown mechanism and growth of osseous tissue at the insertion of the stylohyoid ligament [5].

Symptomatology has various origins. It has been claimed that infraction of styloid process can lead to granular tissue formation thus releasing pressure to nearby structures. Cranial nerves such as glossopharyngeal, vagus and 3^rd ^branch of trigeminal or chorda tympani can also be directly stimulated by the styloid process and induce pain. More reasons for the symptoms include inflammation of tendons, pharyngeal mucosa excitation and impact of carotid bulb [5].

Appropriate choice of therapy, for symptomatic cases, depends on pain intensity or dysphagia and it can be conservative or invasive. These include anti-inflammatory and corticosteroid drugs. If the Symptomatology persists then surgical treatment could be helpful by excision of elongated styloid process [5] but is not advised from some authors [12].

## Conclusion

The importance for early identification of asymptomatic stylohyoid ossification cannot be underestimated. Any overpressure at the surrounding area of tonsilar fossa or violent manipulations around the neck area by medical, paramedical or manual therapists and rehabilitation personnel may lead to fracture, with many clinical subsequences for the patient. In elder patients with undiagnosed neck and/or facial intermittent pain an elongated styloid process could be suspected so further clinical and radiological investigation is advisable.

## Consent

A written consent was obtained by the cadaver's next of kin for publication of the article. A copy of the written consent is available for review by the Editor-in-Chief of this journal.

## Competing interests

The authors declare that they have no competing interests.

## Authors' contributions

GP have collected the finding from the osteological collection of Anatomy Department and supervised the manuscript writing. AR, LL and PK performed the literature review and wrote the draft of the manuscript. AR has obtained the photos. All authors have read and approved the final manuscript.

## References

[B1] Standing S Skull and Mandible Gray's Anatomy. The Anatomical basis of clinical practice 2005 39 Elsevier, Edinburg 470

[B2] Eagle WW The symptoms, diagnosis, and treatment of elongated styloid process Am Surgery 1962 28 1 5 13888940

[B3] Moffat DA Ramsden RT Shaw HJ The styloid process syndrome: aetiological factors and surgical management J Laryngol Otol 1977 91 4 279 294 10.1017/S0022215100083699 856922

[B4] Skrzat J Mroz I Walocha J Zawilinski J Jaworek JK Bilateral ossification of the stylohyoid ligament Folia Morphol 2007 66 3 203 206 17985321

[B5] Murtagh R Caracciolo J Fernandez G CT Findings associated with Eagle syndrome AJNR 2001 22 1401 1402 11498437 PMC7975191

[B6] Gossman FR Jr Tarsitano JJ The styloid-stylohyoid syndrome J Oral Surg 1977 35 555 560 406372

[B7] Ferrario VF Sigurta D Daddona A Dalloca L Miani A Tafuro F Calcification of the stylohyoid ligament: incidence and morphoquantitative evaluations Oral Surg Oral Med Oral Pathol 1990 69 524 529 10.1016/0030-4220(90)90390-E 2326043

[B8] Scaf G Queiroz de Freitas D de Castro Monteiro Loffredo L Diagnostic reproducibility of the elongated styloid process J Appl Oral Sci 2003 11 120 124 10.1590/S1678-77572003000200007 21409324

[B9] Vougiouklakis T Overview of the ossified styloid ligament based in more than 1200 forensic autopsies J Clin Forens Med 2006 13 268 270 10.1016/j.jcfm.2005.09.006 16442338

[B10] Lengele BG Dhem AJ Length of the styloid process of the temporal bone Arch Otolaryngol Head Neck Surg 1988 114 1003 1006 3408565 10.1001/archotol.1988.01860210069018

[B11] Zeliha U Sebnem O Gorkem E Asim A Patel B Elongated Styloid Process and Cervical Spondylosis Clinical Medicine: Case Reports 2008 I 57 64 10.4137/ccrep.s792 PMC3785345 24179348

[B12] Blomgren K Qvarnberg Y Valtonen H Spontaneous fracture of an ossified Stylohyoid ligament Journal of Laryngology and Otology 1999 113 854 855 10664695 10.1017/s0022215100145402

[B13] Johnson CP Scraggs M How T Burns J A necropsy and histomorphometric study of abnormalities in the course of the vertebral artery associated with ossified Stylohyoid ligaments J Clin Pathol 1995 48 637 640 10.1136/jcp.48.7.637 7560170 PMC502714

[B14] Omnell KH Gandhi C Omnell ML Ossification of the human stylohyoid ligament Oral Surg Oral Med Oral Path 1998 85 226 232 10.1016/s1079-2104(98)90431-0 9503461

[B15] Ruprecht A Sasrty KARH Gerard P Mohammad AR Variation in the ossification of the stylohyoid process and ligament Dentomaxillofac Radiology 1988 17 61 66 10.1259/dmfr.1988.0008 3251797

